# Assessing stakeholder’s perception and utilisation of frailty assessment in a vascular surgery setting – a national mixed methods study

**DOI:** 10.1186/s12893-026-03803-5

**Published:** 2026-05-11

**Authors:** S. Welsh, J.K. Burton, D. Orr, T. Quinn

**Affiliations:** 1 Department of Vascular Surgery, Queen Elizabeth University Hospital, Glasgow, 07786123590 UK; 2 School of Cardiovascular & Metabolic Health, College of Medical, Veterinary and Life Sciences, New Lister building, University of Glasgow, Glasgow Royal Infirmary, Glasgow, UK

**Keywords:** Frailty, Vascular Surgical Procedures, Frailty assessment, Service Provision

## Abstract

**Objectives:**

Frailty is common among patients undergoing vascular surgery and is associated with poorer postoperative outcomes. However, the use of frailty assessment tools in vascular services varies considerably. This study explored how vascular healthcare professionals (HCPs) understand frailty and perceive the role of frailty assessment to inform future service development.

**Methods:**

A national mixed-methods study was conducted. A questionnaire captured clinicians’ knowledge, attitudes, and current frailty assessment practices. Semi-structured interviews with consultant and trainee vascular surgeons and other allied healthcare professionals explored experiences in depth. Quantitative data were analysed descriptively; qualitative data were examined using reflexive thematic analysis. Findings were integrated during interpretation.

**Results:**

Survey responses (*n* = 60) showed that frailty is widely recognised as clinically important, yet its assessment is inconsistent, with most respondents (73%) relying on subjective judgement rather than formal tools. Interviews (*n* = 20) identified four overarching themes: (i) conceptualising frailty through a vascular lens; identifying (ii) drivers for change in approach to frailty; the (iii) assessment of frailty by the vascular team was varied but important; and HCPs identified potential risks and benefits to (iv) operationalising frailty for vascular services. Clinicians perceived value in standardising frailty assessment to support early identification, guide optimisation, enhance shared decision-making, and create a common language across specialties. Participants described benefits of collaborative models involving geriatricians, including improved medical management, streamlined discharge planning, and better continuity across hospital–community interfaces. However, barriers included limited time, staffing constraints, uncertainty about tool selection, concerns about service ownership and potential deskilling. Most clinicians felt a multidisciplinary frailty-informed model would ultimately improve outcomes and patient experience if adequately resourced.

**Conclusion:**

Vascular HCPs consider frailty assessment clinically meaningful and potentially transformative, but current practice is inconsistent. Participants perceived strong value in standardising frailty assessment to improve education, awareness and a shared understanding which would promote cross-disciplinary working. Improved health outcomes, patient counselling/education and job role satisfaction were implicated in this movement. However, optimal tool selection and its impact on clinical outcomes remain uncertain and require prospective evaluation before widespread adoption.

**Supplementary Information:**

The online version contains supplementary material available at 10.1186/s12893-026-03803-5.

## Key findings

In this national mixed methods study, sixty questionnaires and 20 interviews were conducted recruiting vascular surgeons and allied health care professionals. Frailty was felt to affect significant numbers of vascular patients. However, it is uncommonly assessed in a standardized fashion which challenges the delivery of comprehensive/holistic care in vascular services with inherent risk of avoidable harm.

## Take home message

A call is made for standardising frailty assessment as part of a service restructuring movement to ameliorate the morbidity of frailty in vascular patients.

## Background

Frailty is a syndrome characterised by increased vulnerability to even minor stressors brought about by an accumulation in age-related deficits across multiple domains [1]. In vascular surgery this is associated with poorer health outcomes, such as a disability, dependence, hospitalisation, prolonged length of hospital stay, morbidity and mortality [2–5]. In addition to these personal costs, frailty is associated with an increased financial burden due to additional care needs [6, 7].

The adoption of Realistic Medicine as a Scottish health policy encourages clinicians to consider personalised approaches to care, manage risks better and reduce patient harm [8]. It also aims to empower patients to discuss their treatment with healthcare professionals (HCPs), prioritising patient wishes and quality of life considerations. The heterogeneity of frailty assessment may challenge professionals’ ability to comfortably deliver realistic medicine.

Frailty has been included in national vascular surgery guidelines where recognising and tailoring treatment for patients living with frailty has clinical and cost benefits [9]. Despite growing academic interest and national guidance, frailty is inconsistently assessed and considered by vascular surgeons in the United Kingdom [10]. Relatively little is known about stakeholders’ perceptions of frailty assessment, how they use frailty tools in decision making, and how services incorporate frailty assessment [10–13]. 

## Methods

### Aim

This study aims to explore HCPs familiarity with, and understanding of, the frailty concept and frailty identification tools within vascular surgery and identify barriers to implementing frailty screening into practice. The study was given favourable ethical opinion from the University of Glasgow, College of Medical, Veterinary & Life Sciences Ethical Committee, Project No: 200,220,006.

### Study design

This is a mixed-methods study. Data were gathered using questionnaires and semi-structured interviews. Original study design was to conduct individual interviews and focus groups. However, during interview processes with multiple interviewees, data collection processes preferentially leant itself to a group interview format. Questionnaires gathered data from a large sample, complemented by qualitative data collection for more detailed exploration. Both aspects are reported in accordance with published guidance [14, 15]. 

### Research team and reflexivity

As primary investigator (PI), SW conducted interviews, managed and analysed data. The author is a female, surgical trainee and conducted the study as part of a postgraduate research fellowship. Through previous clinical experience and attendance at relevant scientific events, the PI had professional relationships with some participants. A second author assisted in thematic analysis, this author is female with a background in academic medicine (geriatric speciality trainee) with a specialist interest in organisation of care services and pathways and published experience in qualitative research methods. Involving a second author with no surgical background was felt useful to ensure rigour and reduce risk of unintentional bias in analysis.

### Participant selection

Any HCP in Scotland with professional experience (current or previous) of working in a vascular surgery service was eligible to participate. Questionnaires were purposively distributed by email. The email list containing clinicians and allied HCP involved in vascular surgery services was generated by contacting regional clinical leads. Representative stakeholders included consultant and trainee vascular surgeons, vascular nurse specialists, clinical scientists, interventional radiologists, staff nurses, consultant anaesthetists and British Association of Chartered Physiotherapists in Limb Absence Rehabilitation (BACPAR)-registered physiotherapists. Non-rotational allied healthcare professionals working in vascular services, such as physiotherapists, occupational therapists and podiatrists were eligible for participation and their recruitment was permitted through snowballing.

Participants were invited to interview sessions through an optional question in the questionnaire, leaving contact details so follow-up sessions could be arranged. Attendees were allowed to invite interested colleagues to interviews, irrespective of questionnaire completion.

### Sample size

No sample size calculation was performed for questionnaire recruitment. Rather, questionnaires were distributed with long response deadlines to encourage uptake. Interview sample size was determined by reaching data saturation, defined as the point in which the PI felt additional data from interviews did not produce new insights based on ongoing iterative analysis without any minimum or maximum number of interviews preferred [16]. 

### Setting

The questionnaire was distributed in November 2022 with a response deadline of April 2023, enabling paper versions to be distributed at the Scottish Vascular Meeting (*a multidisciplinary meeting for all HCPs involved in the delivery of vascular services across Scotland*).

Interviews were conducted between September 2023 – January 2024, primarily virtually (Microsoft Teams, Version 1.0). In-person sessions were offered upon participant preference. Group interviews hosted no more than six participants as this was felt to represent a group size that enables contribution by each attendee gaining a variety of perspectives, whilst being small enough to avoid disorderly or fragmented discussions [17]. Interview groups were organised based on participant availability, grouping participants of similar professional backgrounds to reduce any perceived hierarchical structure to the meeting and encourage participation, e.g., consultant and trainee vascular surgeons were not grouped.

## Data collection

### Questionnaire administration

An 18-item questionnaire on Microsoft Word, Version 2310 was developed in conjunction with a consultant geriatrician (TQ) and piloted on two vascular consultants. It consisted of four parts (participant demographics, perception of frailty significance, frailty assessment methods and implementing frailty assessment into clinical practice) (available on request). Responses were provided through a combination of tick-box answers, Likert scales and free text options. Demographic data answers were categorical to promote anonymisation in the reporting of data. Responses were open for amendment should participants want to alter their answers during completion. Completion of the questionnaire was voluntary and no incentives were offered. Questionnaires were returned to the lead researcher by email or in-person. Hard copies of questionnaires were scanned to create electronic copies for storage before original versions were securely destroyed. Completed questionnaires were electronically stored by participant initials, preventing multiple entries from the same participant, and enabling linkage of data with follow-up interviews. Upon completion of interviews, responses (and paired interview data, where relevant) were transformed to anonymous participant numbers prior to data analysis. Anonymised research data is stored in electronic archives for a period of 10 years in line with the University of Glasgow’s retention policy. The questionnaire was created solely for this study and is included in Supplementary Figure 1.

### Interviews

A semi-structured interview guide was developed and piloted on a consultant geriatrician (TQ) (available on request). In brief, the topics set out to be covered included: exploring participant understanding of the frailty concept, how it might influence clinical practice, methods for assessing and justification for described mode of assessment as well as exploring the perceived role for treating frailty and how best this can be achieved. Virtual interviews were subject to automated transcribing and paired video recording to enable quality control. In-person interviews were conducted in a venue of the participant’s choice and used audio recording and researcher field notes. Microsoft Word, Version 2310 was used for audio transcription and quality control of automated transcribing processes. Participants were not invited to comment on final transcriptions. The interview guide was created solely for this study and is included in Supplementary Figure 2.

### Analysis

All data from questionnaires were entered into a data extraction template using Microsoft Excel, Version 2310. Questionnaires with incomplete responses had areas of missing data coded so that the questionnaire could be included in analysis, excluding only missing data points (sentinel approach). Formal comparative analyses were planned however based on sample size and concerns over multiplicity of testing the decision data is presented descriptively. Free text answers were combined with the interview transcripts and assessed in aggregate. Free text from questionnaires were not used in the quotes, rather they formed part of the development of themes, preferentially reporting quotes from interviews to promote contextualisation of data.

Qualitative data analysis followed Braun and Clarke’s’ six phases of thematic analysis, developing themes from the data [18]. Transcripts were read to enable familiarisation with the content and then coding was undertaken. Codes were then reviewed and organised into specific themes. Within team analysis was undertaken to define and refine theme names, including identification of sub-themes. Identified themes were not returned to participants for comment.

## Results

### Questionnaire results

**Fig. 1:**
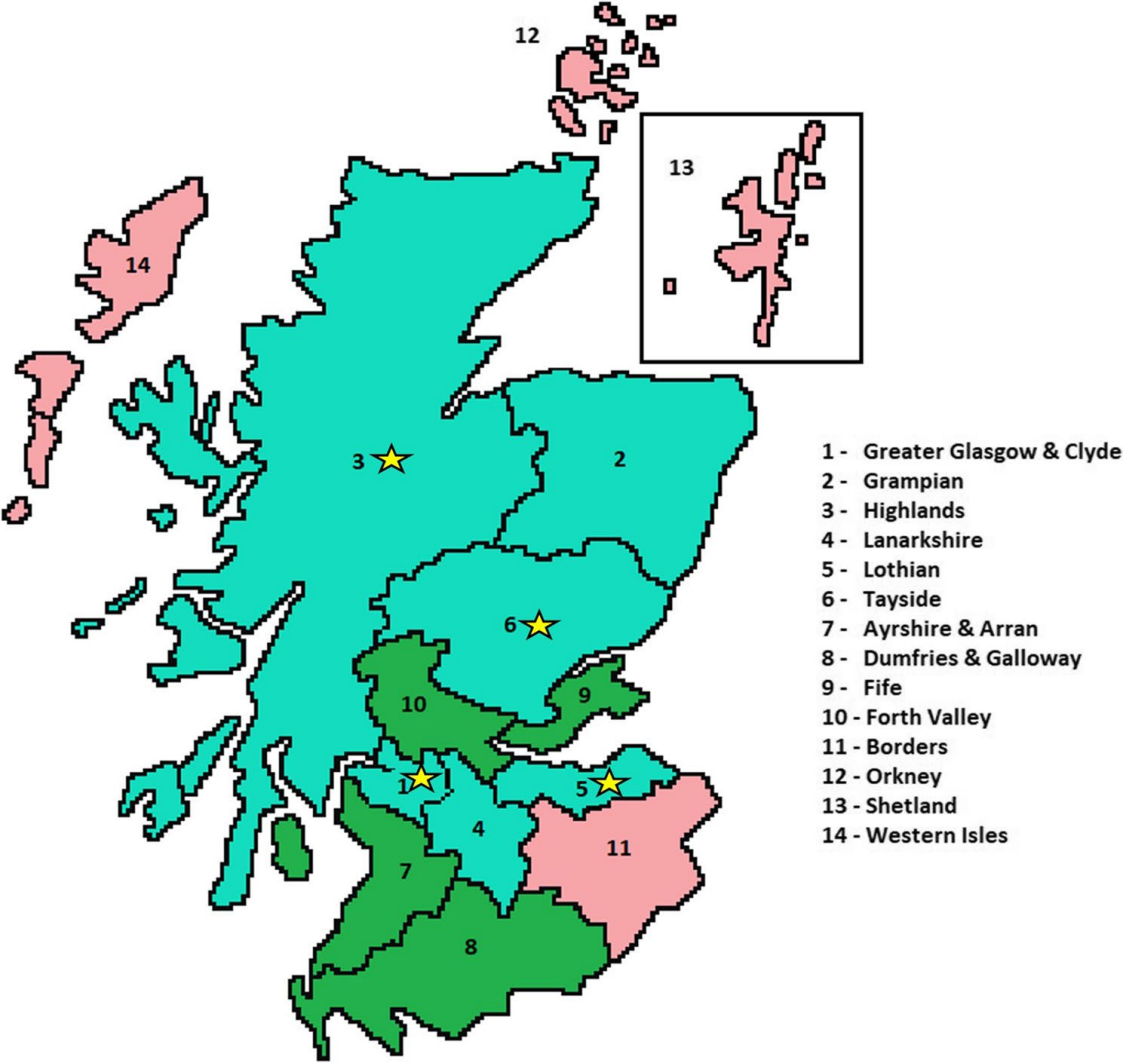
Participating Scottish health boards. This map depicts the 14 regional NHS health boards in Scotland. Green (1–10) indicate health boards that responded to the questionnaire. Pink (11-14) indicate health boards where responses to the questionnaire were not received. Light green health boards (1–6) represent health boards that have a hospital providing a Vascular Surgery ‘Hub’ service. The remaining health boards (7–14) act as vascular surgery ‘spoke’ sites. A gold star indicates health boards that participated in interviews

From 160 HCPs contacted, 60 (38%) returned questionnaires with responses from ten of fourteen Scottish regional NHS health boards, including all six providing a vascular ‘hub’ service (Fig. 1). Completion was excellent with 1181 (99.4%) responses to a total of 1188 questions. Six of seven blank responses asked for free-text.

### Demographics

Questionnaire respondent demographics are summarised in Table 1. Most respondents were male (*n* = 50, 83%), aged 40–49 years (*n* = 19, 32%). There were more responses from surgeons (consultant vascular surgeons, *n* = 24, and surgical trainees, *n* = 17) than non-surgical HCPs (*n* = 19). NHS Greater Glasgow & Clyde returned most questionnaires (*n* = 22, 37%).

**Table 1:** – Characteristics of questionnaire respondents

Sex	Male	50 (83%)
Age (years)	20–29	11 (18%)
	30–39	13 (22%)
	40–49	19 (32%)
	50–59	17 (28%)
	60+	0 (0%)
Years worked in NHS	0–9	21 (35%)
	10–19	13 (22%)
	20–29	15 (25%)
	30–39	11 (18%)
	40+	0 (0%)
Profession	Consultant Vascular Surgeon​	24​ (40%)
	Surgical Trainees (vascular)	17​ (28%)
	Consultant Interventionalist​	5​ (8%)
	Nurse practitioner​	5​ (8%)
	Vascular Nurse Specialist​	3​ (5%)
	BACPAR Physio​	2​ (3%)
	Podiatrist​	1​ (2%)
	Consultant Anaesthetist​	1​ (2%)
	Clinical Scientist​	1​ (2%)
	Scottish Ambulance Service​	1​ (2%)
Scottish regional health board	Greater Glasgow & Clyde (‘Hub’)	22 (37%)
	Tayside (‘Hub’)	10 (17%)
	Lanarkshire (‘Hub’)	9 (15%)
	Lothian (‘Hub’)	6 (10%)
	Grampian (‘Hub’)	5 (8%)
	Highlands (‘Hub’)	2 (3%)
	Forth Valley Royal Hospital (‘Spoke’)	2 (3%)
	Dumfries & Galloway (‘Spoke’)	1 (2%)
	Ayrshire & Arran (‘Spoke’)	1 (2%)
	Fife (‘Spoke’)	1 (2%)
Frequency of assessment (*n* = 60)	Daily	37 (62%)
	Weekly	9 (15%)
	Monthly	2 (3%)
	Rarely	6 (10%)
	Never	6 (10%)
Location of assessment (*n* = 192) (multiple responses permitted; totals exceed *n* = 60)	Outpatient department	43 (22%)
	Acute inpatient admission process	43 (22%)
	Preoperatively	33 (17%)
	Postoperatively	31 (16%)
	In community	16 (8%)
	On the day of surgery	12 (6%)
	Not assessed	10 (5%)
	Other	4 (2%)
Who assesses frailty (*n* = 173) (multiple responses permitted; totals exceed *n* = 60)	Consultant Vascular Surgeon	35 (20%)
	Consultant Anaesthetist	26 (15%)
	Surgical trainee	23 (13%)
	Geriatric physician	22 (13%)
	Ward staff nurse	19 (11%)
	Physiotherapist	18 (10%)
	Occupational therapist	12 (7%)
	Not performed	10 (6%)
	Dedicated surgical frailty team	5 (3%)
	Medical registrar	3 (2%)
Is frailty assessed	Yes	53 (83%)
When frailty is assessed, which assessment tools are used (*n* = 53)	End of bed [19]	44 (83%)
	Rockwood Clinical Frailty Scale [20]	15 (28%)
	Critical Limb Ischaemia Frailty [21]	5 (9%)
	Addenbrookes Vascular Frailty Score [22]	3 (6%)
	Healthcare Improvement Scotland ‘think frailty’ FRAIL scale [23]	3 (6%)
	Grip strength [24]	2 (4%)
	Hospital Frailty Risk Score [25]	1 (2%)
	11-item modified frailty index [26]	1 (2%)
	5-item FRAIL scale [27]	1 (2%)
	Other*	3 (6%)

*Other modes of frailty assessment included: anaesthetic pre-operative assessment (*n* = 1), nursing care assessment such as pressure ulcer risk and nutritional assessment (*n* = 1) and the use of Eastern Cooperative Oncology Group [28] (ECOG) performance status (*n* = 1)

### Approaches to assessment

Most HCP assess frailty (*n* = 53, 88%) and usually do so daily (*n* = 37, 62%). From multiple choice questions, there were 192 responses (from the 60 respondents) to where frailty assessment takes place which is usually in outpatient departments (*n* = 43/192, 22%) or during the acute inpatient admission process (*n* = 43/192, 22%). Surgeons were more likely to assess frailty (*n* = 40/41, 98%) compared to non-surgical HCPs (*n* = 14/19, 74%). Five respondents (all from NHS Tayside) reported the presence of a dedicated surgical frailty team who performed frailty assessments in their unit. Most HCPs do not use a formal tool, instead relying on a subjective ‘End of Bed’ assessment (*n* = 44/53, 83%). If a frailty tool was used, the most popular tool was the Rockwood Clinical Frailty Scale [20] (*n* = 15/53, 28%).

### Significance of frailty

**Fig. 2:**
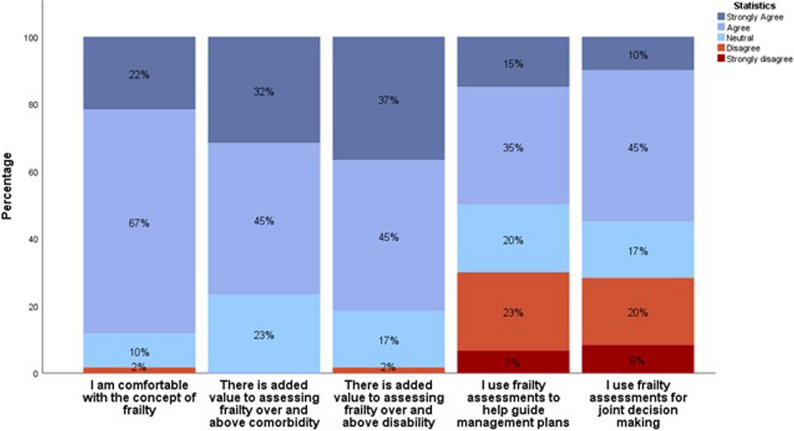
Healthcare providers’ familiarity and utilisation of frailty assessment in clinical practice

Most report being ‘comfortable’ (*n* = 40, 67%) or ‘very comfortable’ (*n* = 13, 22%) with the frailty concept and agree to some extent that there is added value to assessing frailty over and above other constructs of comorbidity (*n* = 46, 77%) and disability (*n* = 49, 82%). A lesser proportion described using frailty assessments to help guide management plans (*n* = 30, 50%) or for joint decision-making (*n* = 33, 55%) (Fig. 2). There were differences between surgeons and non-surgical HCPs. Surgeons were more likely to report being comfortable with the frailty concept (*n* = 40, 98%) compared to non-surgeons (*n* = 13, 68%). Proportionally more surgeons (*n* = 26, 63%) used frailty to guide management plans compared to non-surgical HCPs (*n* = 4, 21%). Consultant and trainee surgeons reported similar practices Supplementary Table 1.

### Interviews

Seven individual interviews and four group interviews (three groups of three and one group of four) were conducted, recruiting 20 participants. Seven of these participants were recruited without questionnaire completion. This included twelve consultant vascular surgeons, three surgical trainees, three vascular nurse specialists, one BACPAR-physiotherapist and one nurse practitioner across four regional NHS health boards with vascular ‘hub’ service provision. A total of 8 h and 4 min of data was recorded (mean duration: 44 min, range 35–60 min). There were no interviews terminated early or cancelled.

**Fig. 3:**
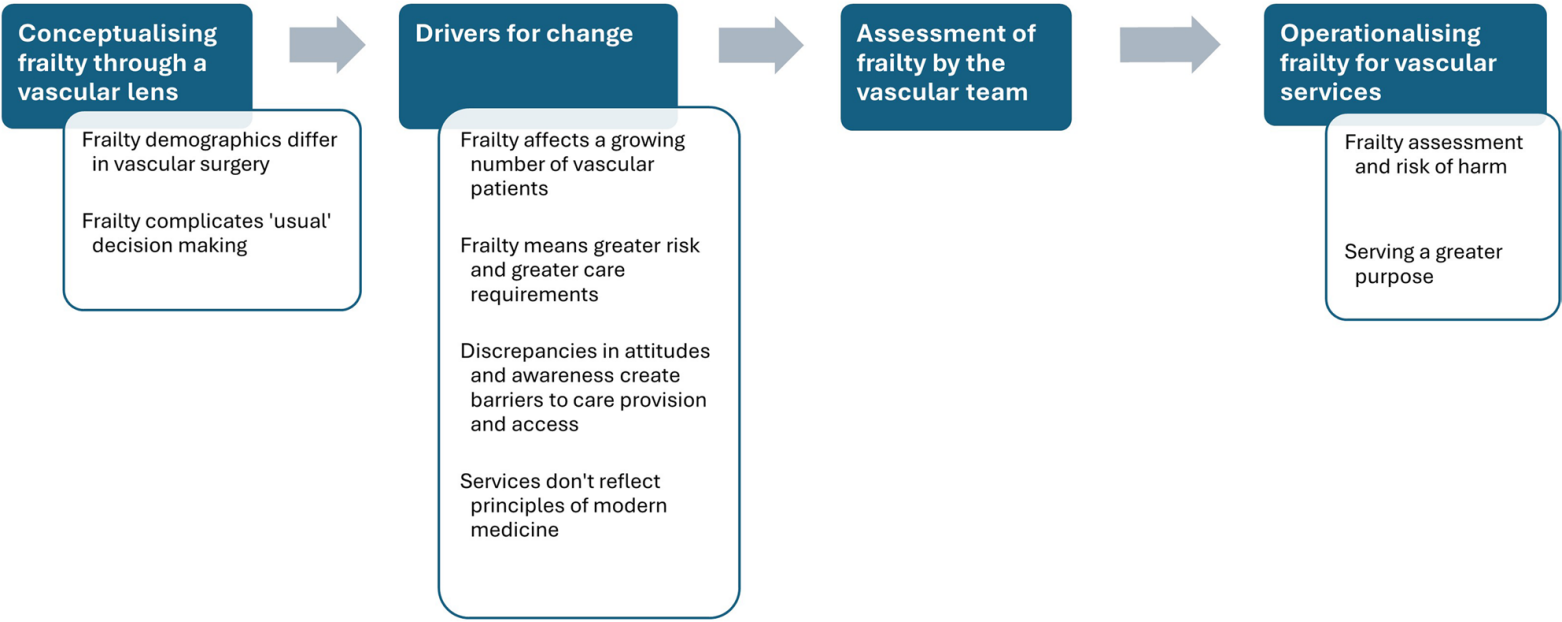
Themes and subthemes

Four themes were developed with several subthemes (Fig. 3). The themes were: (i) conceptualising frailty through a vascular lens; identifying (ii) drivers for change in approach to frailty; the (iii) assessment of frailty by the vascular team was varied but important; and HCPs identified potential risks and benefits to (iv) operationalising frailty for vascular services.

### Conceptualising frailty through a vascular lens

There was agreement around frailty existing as a complex, multidimensional construct, producing a degree of vulnerability and/or dependence which can exist independently from age, comorbidity and physical fitness:

“Frailty covers a huge spectrum of situations where patients have become less physiologically robust…it’s a multifactorial thing which is slightly difficult to define and certainly slightly difficult to quantify as well” (Consultant vascular surgeon, CVS1).

#### Frailty demographics differ in vascular surgery

Frailty was felt to be common in the vascular population. It was appreciated that increasing age is associated with a greater risk of frailty. However, there was a clear feeling that, in a vascular population, there was a disproportionately greater number of younger patients presenting with advanced frailty compared to the general population. In these circumstances some alluded to a relationship between frailty, socioeconomic factors and medical comorbidity:

“The amount of high quality life that people have varies far more by socioeconomic status than by age. I think working in Scotland and with the [Vascular] population that we have, it’s so obvious” (Surgical trainee, ST1).

#### Frailty complicates ‘usual’ decision-making

Frailty was recognised as having important prognostic value, mainly that of increased vulnerability to surgical stress which can influence surgical decision making and expected clinical course. Vascular surgery was described as a speciality where a substantial proportion of treatment is offered on a time-sensitive basis to patients with high disease burden and with a variety of treatment options, each affording a different burden of stress. Clinical decision-making was felt to be intricate and nuanced, and HCPs recognised an additional complexity that frailty introduces to this process:

“You can recognise it but it’s quite hard to pin down as to what it means, what its implications are and what the severity is…it’s not always straight forward.” (CVS2).

### Drivers for change

#### Frailty affects a growing number of vascular patients

All agreed that frailty is becoming more common. Frailty was commonly described as a result of living longer due to medical treatment enabling comorbid survival. For younger frail patients, social factors were recognised as important contributors to poor health quality and early onset frailty:

“It’s a modern epidemic to some extent. We’re living longer and we’re not living better…so there’s all sorts of problems that probably weren’t there in the past”. (CVS2).

#### How frailty affects care delivery

Participants felt that frail patients required more complex and comprehensive care. Surgeons primarily recognise a prognostic role of frailty and how it could be used to determine treatment options. Most surgeons described increasingly offering non-operative treatment to mitigate the risk of poor surgical outcome. Or they may consider major limb amputation in the patients best interest, providing symptomatic relief of arterial rest pain with lower surgical morbidity. However, a proportion of surgeons recognise that whilst frailty may lead to poorer outcomes in a proportion of patients, embarking on carefully considered surgical intervention with the best chance of limb salvage may be appropriate in an attempt to maintain patients’ independence. The latter is a complex decision-making process balancing patients’ robustness, treatment burden and rehabilitative potential against the burden of long term non-operative treatment:

“By salvaging *[arterial reconstruction]* him, I think we will be able to maintain functional independence for him…Whereas, if we were to subject someone with the level of frailty which he currently has to a major limb amputation, I think he would struggle to rehabilitate”. (CVS3).

Nonmedical HCPs reported a loss of independence and reliance on (in)formal support as a key part in the frailty pathway. This meant frail patients typically had increased nursing care needs such as dependence in personal care, nutrition, as well as a greater need for social and emotional support. Delivering additional care needs in a fast paced surgical ward was reportedly challenging with described negative consequences to the general health and rehabilitative capacity of frail patients:

“There are more people that have more needs now, like incontinence, or 2-hourly turns to prevent skin damage…so, if your *[care support workers]* aren’t on the ball, then people can slip through the net. (Staff nurse, SN).

The subjective experience of frail patient’s (and their relatives) experience of care delivery was also reflected upon. There was a consensus that the general population had inconsistencies in their understanding of the frailty concept. A lack of understanding and recognition was associated with unrealistic expectations and the risk of negative experiences of complex health care journeys:

“The biggest challenge perhaps is with patients. Because, well [I know that] some people don’t recognise their own frailty. During COVID there were lots of people who’s *[elderly relative]* were really, really fit and well until they went into that nursing home and died. But they weren’t that fit and well when they go to that nursing home really, they were frail.” (CVS3).

#### Barriers to delivering frailty centric care

While interviewees felt they had a good understanding of frailty and its implications, there was a general impression this was not reflected on a wider service level due to a lack of education and poor awareness. As a result, mobilising necessary resources becomes challenging. This was felt to be a significant barrier for frail patients accessing appropriate care:

“I have worked in units or specialities where clinicians have identified frailty as a reason to disengage or not accept referrals *[due to]* their concern about the patient’s anticipated poor clinical outcomes…”. (ST2)

This is a particular issue in rehabilitation processes and supporting safe transitions between hospital and community care. Growing pressures on community and social care were commonly reported to prolong hospital admissions, resulting in more complex periods of convalescence. The need for clear, bidirectional lines of communication between hospital and community care, in a multidisciplinary capacity, was emphasised to support patient’s ability to access and/or comply with their management:

“Giving anyone a decent class of stocking, if they’re frail, they’re not going to manage so you need to bear that in mind in terms of how they’re going to be supported, either by community nurses or by family or carers or whatever else.” (Vascular nurse specialist, VNS).

#### Services not reflecting principles of modern medicine

A key driver for changing the approach to managing frailty is the professional and responsibility vascular surgeons feel in managing surgical and medical aspects of their patients. Yet increasingly complex patients and a rapidly evolving evidenced-based health care system has made it challenging to deliver such holistic care on an individual level:

“I also think that when you have really complexly comorbid patients, when you’re surgical, it’s difficult to keep on top of all the changes in medicine and what the current best practice is…” (ST1).

Challenges were also felt in a broader sense where part of the success in service delivery relies on rapid patient turnover which does not always align with individualised patient care. There was an appreciation that the service could perhaps be redesigned to better meet the needs of these patients and optimise staff utilisation:

“We’re going back to the health economics thing, what’s the best use of us as vascular surgeons? If we could be freed up by getting help with [medical management of patients], that might be within our remit, but not quite, that would be useful”. (CVS4.)

#### Assessment of frailty by the vascular team

All interviewed surgeons reported assessing frailty as part of clinical interactions with patients. While such an assessment was unanimously described simplistically (‘end of bed’ assessment), the component parts of this process reflect the complex and multidomain nature of the construct. Clinicians described a variable constellation of screening questions, as well as an in-person visual assessment, developed through clinical experience, to establish a combination of functional (in)dependence, medical co-morbidity, cognitive function and psychosocial parameters which were used to ascertain the presence, and severity, of frailty:

“I suspect a lot of my colleagues make a subjective assessment of frailty, looking at physiological reserve, multiorgan dysfunction, physical capability, ability to mobilise, potential to rehabilitate, higher level of function, their independence, need for carers, meal prep…it’s a combination of these things.” (CVS5).

Surgeons unanimously described professional pride and confidence in their clinical acumen, acknowledging subjectivity in assessment without a loss of ‘test’ sensitivity or accuracy:

“I think we don’t routinely use a formal frailty score, but I don’t think that means we notice less frailty.” (CVS6).

Conversely, non-medical HCPs tended to describe a lack of confidence in perceived ability to assess frailty due to an lack of education and structured guidance on how to do so:

“I don’t think I do a good job; I don’t think I properly assess it. I think it’s a lack of confidence, that’s for sure. Just, I have never properly looked at it [assessing frailty], but you know, it’s crossed my mind before. Because I have thought about it, we should probably all look at this in a bit more detail.” (Physiotherapist).

The ability to identify a preferred frailty assessment tool proved difficult with HCPs citing several challenges to this process, including lack of awareness/education, tool unfamiliarity due to the abundance of choice, a lack of evidence and a lack of confidence in tool selection:

“The problem is also you never know what other people’s understanding *[of frailty and frailty tools]* is. (ST2).

Surgeons identified two primary barriers deterring the adoption of objective frailty assessment instruments. First was the ability of a population based tool to conceptualise the decision-making processes that underpin a surgeon’s subjective assessment. Related to this, some expressed concern about potential patient harm such tools could bring if used as a replacement for clinical judgement, influencing patient selection and introducing unnecessary additional paperwork to time-limited clinical situations:

“So, I think we make a complex, multifaceted decision…I need *[that]* replicated *[in an assessment tool]* because clearly, I don’t want to overtreat people, but I also don’t want to undertreat people.” (CVS4).

Secondly, there were concerns around the utility of frailty tools for prognostication. Surgeons felt for most accurate prognostication the instrument would need to consider disease and treatment-specific burden. Yet this would produce an abundance of tools which would deter uptake. However, it was accepted that frailty assessment likely plays a bigger role than solely acting as a predictive test and for this reason there was merit in standardising frailty assessment, even beyond vascular surgery specialty. It was felt standardisation would endorse a universal understanding of the frailty construct, as well as the proposed assessment format, which would be key to setting up and delivering frailty-centric care:

“Having a single standardised frailty assessment tool across NHS Scotland in clinical practice would be helpful, even if the prognostic accuracy is less in certain circumstances.” (ST2).

The desired tool characteristics of a proposed standardised tool were explored with surgeons and have been included in Supplementary Figure 3. These included relevant, accessible, valid, evidenced and standardised.

### Operationalising frailty for vascular services

**Fig. 4:**
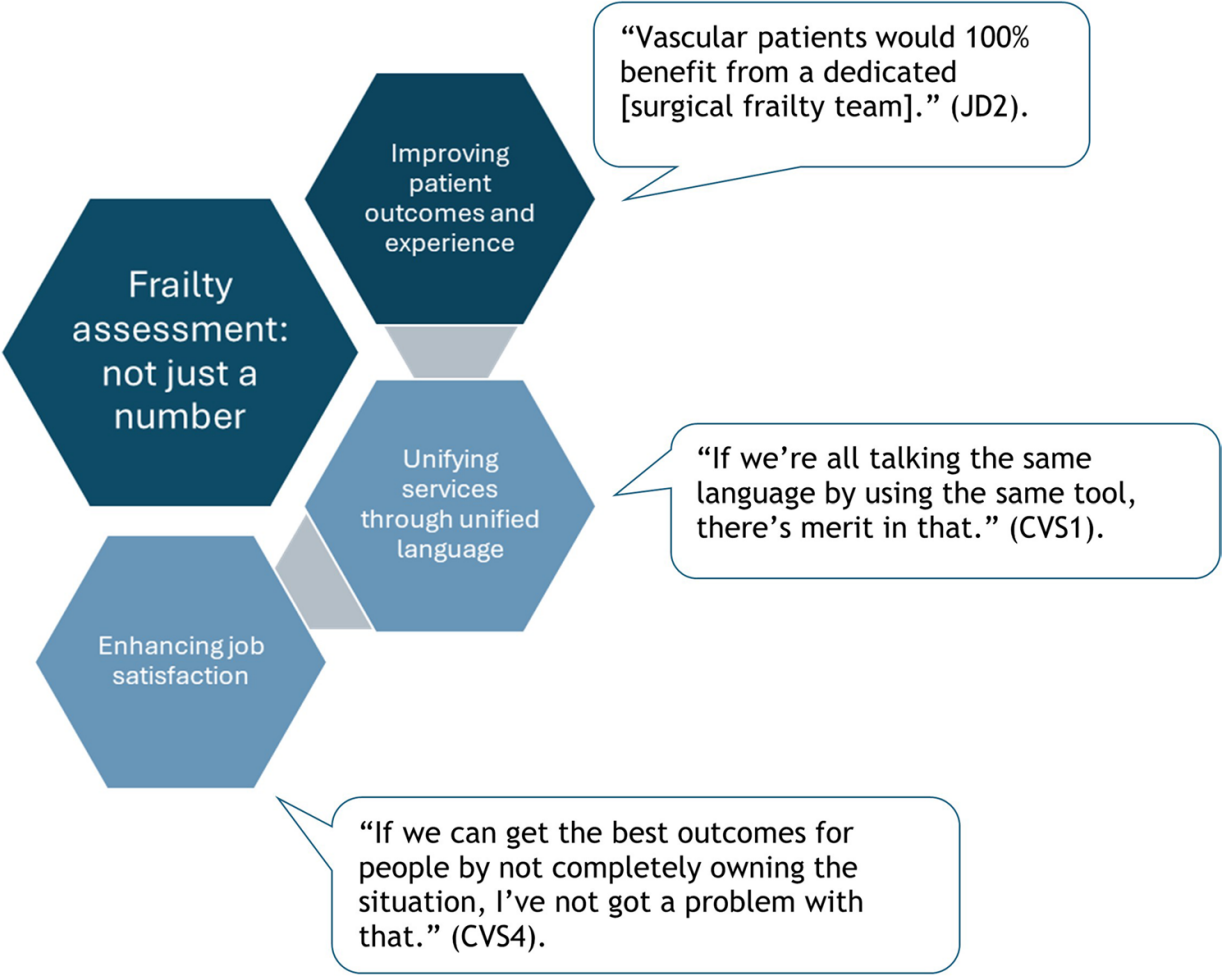
**-** Frailty assessment serving a greater purpose

HCPs described value in formalising frailty assessment beyond numerical scoring. They felt more could be done to address the multifactorial nature of frailty through early identification, multidisciplinary collaboration and shared understanding. Introducing a standardised assessment process was perceived as a useful foundation for this, with three key benefits identified: improving patient outcomes, unifying services through shared language, and enhancing job satisfaction (Fig. 4).

#### Improving patient outcomes and experience

Standardised frailty assessment was viewed as a way to enable *proactive rather than reactive* care. Participants felt earlier recognition of vulnerability could support timely multidisciplinary input:

“I think to really benefit you would need to be almost prospectively identifying these patients. Because again, I think one of the things we do less well on a surgical ward is that we get medical help quite late for these patients.” (ST1).

Frailty screening was seen as means of identifying patients for quantification of their multidisciplinary needs and enable tailored medical, nursing and social care, focusing on aspects of optimisation that reduce perioperative risk and enhance recovery. Although most discussion centred on acute presentations, some saw potential for pre-operative optimisation in elective cases, albeit with the caveat that evidence on reversibility remains limited:

“There isn’t enough evidence around reversibility of frailty…however optimising deficits would be good for a select group.” (ST3).

Frailty assessment was also valued for supporting shared decision-making and managing expectations:

“Frailty assessment helps informed discussions with patients about the likely impact of surgery and realistic recovery.” (CVS7).

#### Unifying services through shared language

Despite recognising the multidisciplinary nature of frailty, participants described current care as disjointed. A standardised frailty tool was viewed as a mechanism to create a *shared language* across disciplines, enhancing communication and continuity, Table 2:

**Table 2:** Perceived service-level benefits of standardised frailty assessment

Domain	Perceived benefit
Clinical care	Earlier identification, targeted optimisation, and improved decision-making
Team communication	Shared language across specialties and settings
System efficiency	Reduced duplication, improved discharge coordination, clearer referral pathways
Patient experience	Better-informed expectations, smoother transitions of care

“There’s merit in extending this beyond vascular — if we’re all talking the same language by using the same tool, there’s merit in that.” (CVS1).

To support delivery of Realistic Medicine vascular surgeons generally recognised a role for involving geriatricians, as frailty experts, in elements of patient care. Proposed roles included proactively managing nuanced medical care, providing a medical opinion on likely post-operative trajectory, bringing close links with related medical specialities and improving efficiency in complex discharge planning process through familiarity with relevant in-hospital allied healthcare professionals and relevant spoke and community services.

“It would be very good to have more dedicated medical input — our patients are all old and frail, and geriatricians are probably the right people.” (CVS6).

Collaborative models were also perceived to improve overall service functioning by clarifying responsibilities, reducing duplication of workload, improving efficiency in workload and time management and enhancing access to support services — particularly important after centralisation of vascular services:

“*[The surgical frailty team are]* has created really good links between in-hospital medical management and community care which helps expedite discharge planning. (CVS7).

A loss of continuity in primary care, workforce shortages, difficulties in accessing services and non-medical HCP referral pathways were perceived to have altered the style of referrals. Consequently, triaging community and hospital referrals can be more challenging and while a frailty score could not replace clinical acumen, it was felt it would help enhance awareness of the construct, enhance patient counselling and increase opportunities for delivering Realistic Medicine:

“GP’s have a different way of working now where they don’t see the same patient or have continuity the same so, absolutely, having a frailty assessment in the GP letter would be helpful to increase the amount of detail on the referral.” (CVS10).

However, workforce shortages, limited funding and competing priorities were viewed as significant barriers to sustaining such services. Participants acknowledged that demonstrating measurable benefit would be key to maintaining investment:

“We used to have a frailty service, but it was withdrawn because it wasn’t seen as cost-effective — though we thought it was good.” (CVS10).

#### Enhancing job satisfaction

Where implemented, collaborative vascular–geriatric models were viewed positively. Participants described mutual learning and greater confidence in managing complex patients:

“It’s a positive environment — both teams feel valued, and patients benefit.” (CVS7).

Integrated geriatricians with vascular surgical teams empower vascular HCPs in caring for frail patients and was coupled with a sense of satisfaction from the perception frail patients were better cared for:

“[Having a frailty team] gives me confidence in escalating concerns. Outcomes are better because of their different background and skillset.” (Physiotherapist).

Some surgeons, however, expressed concern about losing patient ownership and a proposed resultant deskilling in clinical decision making which may not be complemented by improvements in surgical skills:

“My worry is that the healthcare service isn’t set up for efficient theatre utilisation so if we are not involved in patient care outside of theatre, then job satisfaction may drop substantially. We will just be sitting around as surgeons…” (ST3).

Acknowledging these issues, the common priority expressed by surgeons was ensuring appropriate, tailored and patient-centred care to reduce harm, improve health outcomes and increase patient satisfaction. For most, the understanding was that these goals were more likely to be successfully met through a collaborative multidisciplinary approach:

“I would value a geriatrician’s opinion. I think the key thing is not about ownership but it’s about collaboration. If we can get the best outcomes for people by not completely owning the situation, I’ve not got a problem with that.” (CVS4).

## Discussion

This study set out to address an evidence gap, assessing the perception and utilisation of frailty assessment by Scottish vascular surgery HCPs. Improved understanding is critical to develop approaches to encourage the up-take and roll-out of collaborative frailty-centric pathways and bring practices in line with national guidance.

Frailty is recognised by most participants as an important construct due to its association with complex care needs and poor health outcomes. It was recognised by HCPs that services could be better structured to ameliorate this morbidity. Despite this, the questionnaires demonstrate a discrepancy between appreciation of frailty as a clinically relevant construct and the frequency to which frailty assessment is used in clinical decision-making. This appears to be due to unfamiliarity with assessment tools, lack of awareness/education and disjointed service delivery impeding the delivery of comprehensive care. Standardising frailty assessment was felt to introduce a common language around frailty which would help overcome some of these barriers. Surgeons primarily considered frailty in terms of patient selection for treatment. Concerns around the ability of a tool to conceptualise complex clinical assessment meant surgeons were less inclined to use objective measures of frailty. In keeping with current literature, care providers report a surplus of validated tools causes unfamiliarity, confusion and a concern over tool validity [10]. In looking beyond this however, surgeons recognised that evolving patient demographics and restricted resources increasingly challenged holistic patient care. A growing role for integrated multidisciplinary team input for frail patients was identified. Subtle differences in opinions between consultant and trainee surgeons were apparent. Trainee surgeons were more inclined to report an unfamiliarity with the complexities of frailty and a preference for involving frailty specialists. Comparatively, consultants generally were more comfortable with these complexities. Despite this, a role for involving geriatricians was generally agreed upon as this would not only benefit patient care but also facilitate service planning, creating more time for surgeons to utilise their skillset on surgical care.

Non-surgical HCP generally viewed frailty more holistically. Value is seen in early frailty identification to assist in managing aspects of the whole patient journey, identifying those with greater care and discharge planning requirements. However, there is an apparent lack of confidence around frailty assessment and an appetite for more formalised training to assist in recognising, and communicating, its presence.

Where historic research efforts primarily propose the role of frailty assessment for prognostication purposes [29], perhaps the greatest benefit may sooner be observed in unifying language, improving awareness and creating shared goals in frailty management. This will help overcome barriers to improving outcomes by guiding the restructuring of disjointed and resource-limited healthcare services, ultimately enabling infrastructure to better reflect the principles of modern healthcare which HCPs strive toward. Unifying health and social care services’ approach to caring for vascular patients with frailty is of critical value following the centralisation of vascular services where the successful delivery of medical and nursing care relies on clear, concise communication between all members of the multidisciplinary team caring for vascular patients, across community care, primary, secondary health care centres and between hub and spoke services [9]. Key to this is the proposed continuous collaborative efforts between geriatricians and care providers with a specialist interest in care of the older patient with surgical directorates [30]. 

Geriatricians are similarly incentivised by a collaborative approach due to a recognition that vascular patients in particular often have considerable care needs that could most suitably be met by a geriatrician’s skillset [31, 32]. In recognition of shared priorities there is growing momentum in establishing integrated perioperative services [33]. The British Geriatrics Society has produced guidelines setting out key clinical, governance and educational/training principles for service models to refer to when setting up collaborative pathways: Peri-operative Care for Older Patients Undergoing Surgery (POPS) [12]. A key recommendation within these guidelines include:

“All patients aged over 65 years, and younger patients at risk of frailty, referred for elective or emergency surgery, should have frailty status documented at referral, preoperative assessment and admission using the Clinical Frailty Scale (CFS).”

Examples of successful alignment of services has produced a strong business case for involving geriatricians in the care of surgical patients [33–36]. Demonstrable improvement in health outcomes across vascular surgery and non-vascular surgical specialities alike have been observed and these improve as models evolve over time [35, 37]. Pathophysiological, accelerated ageing processes also exist in chronologically younger vascular patients (aged < 65 years). Aetiological risk factors have been identified for early onset frailty, such as socioeconomic deprivation, multimorbidity, homelessness, substance misuse, smoking, and urban residence which are also strongly associated with vascular disease [38, 39]. For this reason, recent NHS England guidance propose a role for involving geriatricians in the care of younger patients suffering from early onset frailty; this will promote patient centred care for vulnerable patient populations and ensure equity healthcare delivery [40]. 

### Consistency with frailty assessment in current literature

The data generated by this study is consistent with the previously mentioned UK [10] study as well as a national Italian cross-sectional study of vascular surgeons’ perception and utilisation of frailty assessment [41]. Italian vascular surgeons demonstrated a high level of appreciation of the importance of frailty as a construct (93%). Despite this, most surgeons do not use a formal frailty assessment instrument due to insufficient guidance on available instruments as well as a relative knowledge gap around perioperative management of frailty, and a lack of staff or time. Of note, when frailty assessment tools were used, the Rockwood Clinical Frailty Scale (CFS) [20] was also preferred. It is therefore likely that frailty in vascular surgical populations is commonly underassessed and likely undertreated on an international scale, emphasising the importance of unifying language around this important construct.

### Strengths and limitations

This was a national mixed methods study with excellent recruitment from most Scottish health boards, including all that provide a vascular surgery ‘hub’ service. Including all members of the clinical team caring for a vascular patient was a deliberate effort to capture frailty and its implications on a vascular service in the broadest sense. The broad inclusion and data collection method enables this study to recognise the far-reaching effects of frailty for all relevant care providers within vascular services. This may better inform policy makers and any subsequent implementation of frailty care programs for vascular patients in secondary care.

A key challenge lies in assessing healthcare professionals’ understanding of a construct that is neither universally defined nor consistently evaluated. This raises the possibility that interviewees may be discussing frailty syndrome inaccurately, potentially conflating it with related or distinct constructs. The aim of this study was not to evaluate clinicians’ definitions of frailty against a reference standard, as no universally accepted definition currently exists. Rather, the observed lack of a clear and consistent definition among clinicians constitutes a key finding. Frailty is commonly conceptualised as an age-associated syndrome characterised by multidomain deficits; however, variation in the domains affected across individuals contributes to heterogeneity in its clinical presentation [42]. This complexity is compounded by the absence of a gold standard definition, which has led to the generation of an abundance of heterogeneous frailty assessment tools: a total of 43 methods of frailty assessment has been identified in vascular research and clinical practice [29]. The perceived benefit to standardising frailty assessment reported by participants in this study were in bridging these discrepancies in understanding by unifying language and conceptual understanding. This would allow a framework against which integrated services can be developed thereby streamlining access to services where needed. The implementation of such a standardised assessment tool would require prospective validation. The inclusion of expertise from frailty specialists in this process would be beneficial. Of course standardising frailty assessment or developing clear guidelines around its assessment should not replace the clinical acumen of experienced clinicians. Instead the tools/service restructure would sit alongside clinical practice, enhancing access to relevant services where required.

Vascular surgeons made a significant contribution, with data saturation felt to have been satisfactorily achieved. A role for expanding on the views of allied HCPs is identified to accurately reflect the multidisciplinary care team that is required to provide holistic care for frail surgical patients. Where allied HCPs did take part, there was a self-described lack of confidence on the topic, reported to be due to inadequate training provision, which may have negatively impacted recruitment.

The professional relationship that the PI had with some of the interviewees means some participants may have presumed a degree of shared knowledge, influencing the content of discussion. The effect of this was minimised through the use of the standardised interview guide. Further, any subsequent unintentional bias in thematic analysis was mitigated through independent coding and joint thematic analysis with an expert in qualitative methodology with no surgical background.

Lastly, the pool of respondents was finite and although response rate was good, the numbers were modest and did not allow for formal significance testing. Thus, interpretations of the quantitative data are based on absolute values only and conclusions need to be mindful of this.

## Implications for future work

The findings in this study resonate with current literature, re-iterating the call for reaching a consensus on a common approach to frailty assessment [43]. This study identifies a role for further research to be targeted at comparing clinimetric properties of subjective means of frailty assessment (“end-of-bed” test) with validated tools.

Despite the growth in geriatric-surgical care models, there remains considerable variation in access across the UK [44]. The structure of various collaborative approaches will differ between healthcare establishments and so the accumulation of dedicated cost-efficiency data as well as the dissemination of experiential knowledge and collaboration with commissioners is sought after to support the continued growth of such services. Where collaborative services have been established, it is worth noting that a minority receive funding from a surgical directorate, suggesting more could be done to improve awareness of the role for geriatric input across surgical boards [45, 46]. 

It is therefore exciting to observe ongoing research exploring the implementation of POPS services across the NHS. The POPS Scale Up (POPS-SUp) study has been specifically designed to assess whether such services can be implemented at scale to improve patient outcomes in a cost-effective way. The project involves traditional outcome measures such as length of stay and postoperative morbidity but also directly involves patients and carers to explore more novel outcome measures such as subjective experience of the pathway and quality of life parameters. This will inform on whether POPS Services can be rolled out across the NHS, how patients might benefit and any potential financial advantages might be brought about by this service adaptation [47]. 

## Conclusion

Vascular surgery HCPs recognise the importance of frailty in clinical practice. Assessment of frailty is primarily performed through subjective clinical assessment as part of any clinical interaction. A call was made for standardising the assessment of frailty, primarily due to a multitude of positive implications this would bring. Primarily improving education and awareness which would support cross-disciplinary working and promote the integration of disjointed services into the multidisciplinary collaborative approach frail patients require from health care services. Improved health outcomes, refining patient education/counselling and enhanced job satisfaction were implicated in this movement. The utility of a standardised frailty assessment in prognostication is disputed by surgeons due to concerns around how a population-based tool can replicate nuanced surgical decision-making and some scepticism in the accuracy around tool prognostication. This study highlights the need for future research to be directed at identifying a standardised tool for service wide adoption, delineating the opinions stakeholders have on desired characteristics for such a tool as well as the need to validate this against the commonly utilised ‘end of bed’ test, as well as the recommended CFS, to infer test validity and encourage uptake in clinical practice. 

## Supplementary Information

Supplementary Material 1: Supplementary Figure 1 – Research questionnaire.

Supplementary Material 2: Supplementary Figure 2 – Interview guide.

Supplementary Material 3: Supplementary Figure 3 – Desired frailty assessment tool characteristics.

Supplementary Material 4: Supplementary Table 1 – Interprofessional differences in practice.

## Data Availability

Pseudonymised datasets used and/or analysed during the current study are available from the corresponding author on reasonable request.

## Abbreviations

BACPAR: British Association of Chartered Physiotherapists in Limb Absence Rehabilitation
CFS: Clinical Frailty Scale
CVS: Consultant Vascular Surgeon
HCP: Health Care Professional
NHS: National Health Service
PI: Primary Investigator
POPS: Peri-operative Care for Older Patients Undergoing Surgery
POPS-Sup: POPS-Scale_Up
ST: Surgical trainee
UK: United Kingdom
