# Supplementary material for: Assessing stakeholder’s perception and utilisation of frailty assessment in a vascular surgery setting – a national mixed methods study
Source: BMC Surg. 2026 May 11;26:448. doi: 10.1186/s12893-026-03803-5 (PMC13340002; doi:10.1186/s12893-026-03803-5)
Supplement: Supplementary file 1 — Supplementary Material 1: Supplementary Figure 1 – Research questionnaire. [file 12893_2026_3803_MOESM1_ESM.pdf]

University of Glasgow | College of Medical, Veterinary & Life Sciences

CIRCULATION FOUNDATION The Vascular Charity

ROYAL COLLEGE OF PHYSICIANS AND SURGEONS OF GLASGOW

Assessing Frailty in Vascular Patients

Thank you for taking the time to complete this survey. Please take the time to read through each question before giving an answer. The survey should take between 10 – 15 minutes.

1. DEMOGRAPHICS

Please tick the relevant box or fill in the blank boxes for each question.

a. Sex — Male ☐ Female ☐ Prefer not to say ☐

b. Age (years)

20 - 29 ☐ 30 – 39 ☐ 40 - 49 ☐
50 – 59 ☐ 60 – 69 ☐ ≥ 70 ☐
Prefer not to say ☐

c. Years worked for the NHS

0 – 9 ☐ 10 – 19 ☐ 20 – 29 ☐
30 – 39 ☐ 40 – 49 ☐ ≥50 ☐
Prefer not to say ☐

d. Profession

Consultant Vascular Surgeon ☐
Consultant Anaesthetist ☐
Consultant Interventionalist ☐
Junior doctor (including non-training grades) ☐
Nurse practitioner ☐
Podiatrist ☐
Occupational therapist ☐
Physiotherapist ☐
Prefer not to say ☐
Other *(please specify)*:

e. Which health board/trust do you work in?

2. SIGNIFICANCE OF FRAILTY

Please circle the relevant number for each answer

	Strongly disagree	Disagree	Neutral	Agree	Strongly Agree
**a. I am comfortable with the concept of frailty**	1	2	3	4	5
**b. I use frailty assessments to help guide management plans**	1	2	3	4	5
**c. I use frailty assessments for joint decision making**	1	2	3	4	5
**d. There is added value to assessing frailty over and above disability**	1	2	3	4	5
**e. There is added value to assessing frailty over and above multi-morbidity**	1	2	3	4	5

3. FRAILTY ASSESSMENT

Please tick the relevant box as appropriate. More than one box may be ticked.

a. When patient-facing, I assess frailty…

- Daily ☐
- Weekly ☐
- Monthly ☐
- Rarely ☐
- Never ☐

b. When in the patient journey is frailty assessed

- In community prior to referral ☐
- In outpatient department ☐
- Pre-operative assessment clinic ☐
- On acute admission ☐
- On the day of surgery ☐
- Post-operatively ☐
- Frailty is not assessed ☐
- Other (*please specify*):

c. Which frailty assessment tools do you use, if any?

- Rockwood Clinical Frailty Scale ☐
- 11-item Modified Frailty Index ☐
- 5-item modified Frailty Index ☐
- Risk Analysis Index ☐
- Edmonton Frail Scale ☐
- Critical Limb Ischaemia Frailty (CLI Frailty) ☐
- Addenbrookes Vascular Frailty Score ☐
- Healthcare Improvement Scotland Frailty Tool ☐
- I do not assess frailty ☐
- Groningen Frailty Index ☐
- Ruptured Aneurysm Frailty Score ☐
- FRAIL Scale ☐
- Electronic frailty index ☐
- Hospital Frailty Risk Score ☐
- Geriatric Nutritional Risk Index (GNRI) ☐
- 'End of bed test' ☐
- Grip strength ☐

Other, or if you do not routinely assess frailty currently, which tool would you use? (*please specify*):

d. In your unit, who (also) performs a frailty assessment

Consultant Vascular Surgeon		Geriatric/MOE team	
Anaesthetic team		Physiotherapist	
Junior doctors (any grade)		Medical registrar	
Ward staff nurses		Occupational therapist	
Dedicated surgical comprehensive geriatric team		No frailty assessment performed	

Other (*please specify, or if no frailty assessment is performed, who do you think should perform it?*):

4. IMPLEMENTING FRAILTY ASSESSMENT INTO CLINICAL PRACTICE

'The Provision of Vascular Services for People with Vascular Disease (2021)' identified the vascular patient population as having a high proportion of frail patients. The following questions pertain to these guidelines. *Please give a short summary for each question. If not applicable, please indicate with: N/A and give a short explanation.*

**a. Does identifying frailty influence your practice? If so, how?**
**b. Do vascular patients in your unit have access to a comprehensive geriatric assessment (CGA) by a suitably trained specialist to address issues of frailty and multi-morbidity peri-operatively?**
**c. Do Care of the Elderly and Frailty members participate in your vascular MDT?**
**d. Do you think there are barriers to implementing frailty assessment in to your clinical practice, if so what are they?**

This is the end of the questionnaire, thank you for taking the time to complete!

If you are interested in participating in a focus group to further discuss frailty and its assessment, please indicate so by leaving contact details in the box below so that we can be in touch with further details in due course (e.g., e-mail/telephone number).

