# Supplementary material for: Assessing stakeholder’s perception and utilisation of frailty assessment in a vascular surgery setting – a national mixed methods study
Source: BMC Surg. 2026 May 11;26:448. doi: 10.1186/s12893-026-03803-5 (PMC13340002; doi:10.1186/s12893-026-03803-5)
Supplement: Supplementary file 2 — Supplementary Material 2: Supplementary Figure 2 – Interview guide. [file 12893_2026_3803_MOESM2_ESM.pdf]

University of Glasgow | College of Medical, Veterinary & Life Sciences
Royal College of Physicians and Surgeons of Glasgow
Vascular Society
The Vascular Society for Great Britain and Ireland
Circulation Foundation
The Vascular Charity

Frailty in Vascular Disease

Interview/Focus group

Miss Silje Welsh
MRCS, MSc, MBChB, BSc (Hons)
Clinical Research Fellow

Primary supervisors: Prof T Quinn & Mr D Orr

1

University of Glasgow | College of Medical, Veterinary & Life Sciences
Royal College of Physicians and Surgeons of Glasgow
Vascular Society
The Vascular Society for Great Britain and Ireland
Circulation Foundation
The Vascular Charity

Purpose of interview

- Follow on from questionnaires
- What role it plays in patient management
- Approaches to assessment
- Frailty-centric service models – ideas for adaptations?

2

Why frailty?

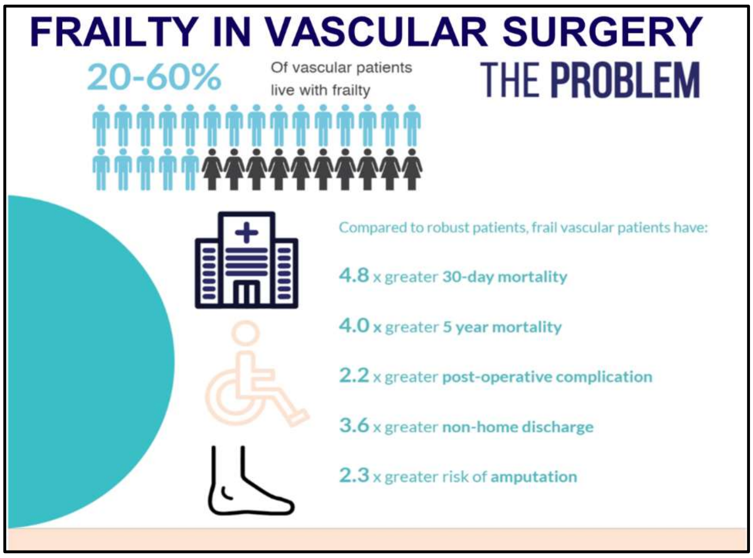

SO WHAT?

Welsh SA, M.P., Pathmanathan S, Hussey K, Brittenden J, Orr DJ, Quinn T, *Frailty in peripheral arteiral disease.* J Vasc. Soc. G. B. , 2023. **2**(3): p. 128-133.

3

Improving health care outcomes

- Identifying a problem
- Communicate the importance of the problem
- Agree the problem is important
- Identify solutions/adaptations
  - Frailty assessment
  - Frailty management: Frailty-centric service adaptations

4

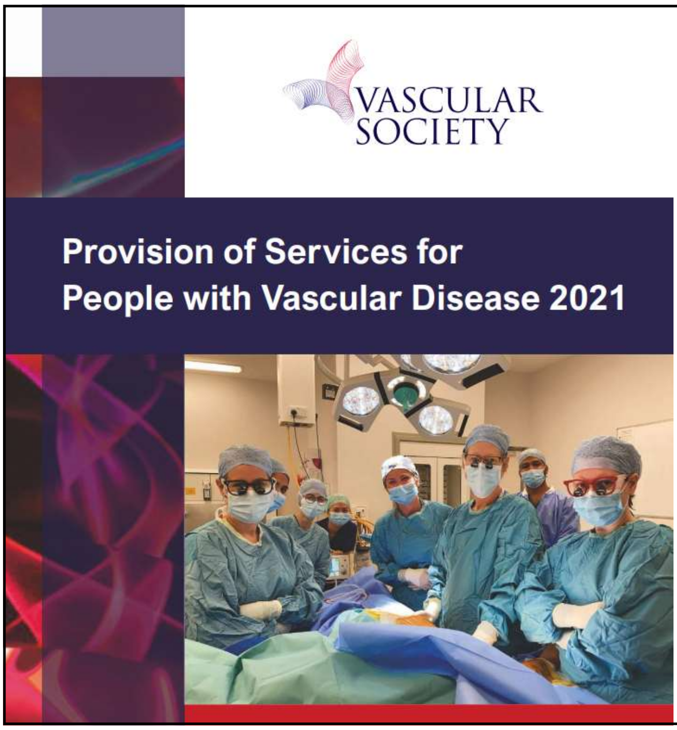

5.22 The importance of frailty, over and above age, is recognised as predicting worse outcomes and longer hospital length of stay after vascular surgery.[6]

2.9 To be both clinically and cost-effective vascular services should deliver the care that their patients want, and need:

- The age of people being treated for vascular disease is increasing, as is the complexity of their disease
- Even 'young' vascular patients (<60 years) can be frail or multi-morbid

Peri-operative care

2.24 Much work has already been done by the Royal College of Anaesthetists and its partners to improve peri-operative medicine.[13]

2.25 VASGBI have produced guidance for both healthcare professionals and for patients on getting the best outcomes from vascular surgery.[14-15]

2.26 People with vascular disease should have access to a comprehensive geriatric assessment (CGA) by a suitably trained specialist to address issues of frailty and multi-morbidity both before and after they have vascular surgery.[16]

Comprehensive geriatric assessment

6.36 In vascular patients age is not an absolute indicator of need, medical specialists with an interest in the elderly and frail have a pivotal role in using CGA to:[11-13]

- Assess risks of surgery
- Support shared decision making
- Guide pre-operative optimisation
- Advise on post-operative management

1. The Vascular Societies of Great Britain and Ireland. *Provision of Services for People with Vascular Disease 2021*. 2021 [Date Accessed:23/08/2023]; Available from: https://www.vascularsociety.org.uk/_userfiles/pages/files/Resources/FINAL%20POVS.pdf.

5

University of Glasgow | College of Medical, Veterinary & Life Sciences — Royal College of Physicians and Surgeons of Glasgow — Vascular Society — Circulation Foundation

Improving health care outcomes

- Identifying a problem
- Communicate the importance of the problem
- Agree the problem is important *OR NOT?*
- Identify solutions/adaptations
  - Frailty assessment
  - Frailty management: Frailty-centric service adaptations

6

How important is frailty to your practice/within your department?

Is there recognition of frailty in your unit / how? (?existence of a frailty service).

Do you think there are ways to improve awareness of frailty? (barriers)

What would be the perceived benefits of detecting frailty?

Does the recognition of frailty translate into change in clinical practice/patient management?

What factors guides decision making for suitability for surgery, how big a role does frailty play and why?

University of Glasgow | College of Medical, Veterinary & Life Sciences
ROYAL COLLEGE OF PHYSICIANS AND SURGEONS OF GLASGOW
VASCULAR SOCIETY
CIRCULATION FOUNDATION The Vascular Charity

Improving health care outcomes

- Identifying a problem
- Communicate the importance of the problem
- Agree the problem is important
- Identify solutions/adaptations
  - Frailty assessment
  - Frailty management: Frailty-centric service adaptations

University of Glasgow | College of Medical, Veterinary & Life Sciences
ROYAL COLLEGE OF PHYSICIANS AND SURGEONS OF GLASGOW
VASCULAR SOCIETY
CIRCULATION FOUNDATION

Frailty assessment (1)

Is there a structured/standardised approach to assessing frailty in your unit?

Positives v negatives of mode of frailty assessment?

Why do you assess frailty in the way that you do? How did you come across selected tools?

Do you know of any others? Reasons for not adopting alternate tools?

University of Glasgow | College of Medical, Veterinary & Life Sciences
ROYAL COLLEGE OF PHYSICIANS AND SURGEONS OF GLASGOW
VASCULAR SOCIETY
CIRCULATION FOUNDATION

Frailty assessment (2)

Would you use different tools for outpatient/elective v inpatient/urgent?

Opinion on disease/treatment-specific tools v generic ones?

Do you commonly assess other prognostic markers ?P-Possum or ASA?

Do you think it would be useful to incorporate frailty into anaesthetic risk stratification tools?

University of Glasgow | College of Medical, Veterinary & Life Sciences
ROYAL COLLEGE OF PHYSICIANS AND SURGEONS OF GLASGOW
VASCULAR SOCIETY
CIRCULATION FOUNDATION

Improving health care outcomes

- Identifying a problem
- Communicate the importance of the problem
- Agree the problem is important
- Identify solutions/adaptations
  - Frailty assessment
  - Frailty management: Frailty-centric service adaptations

11

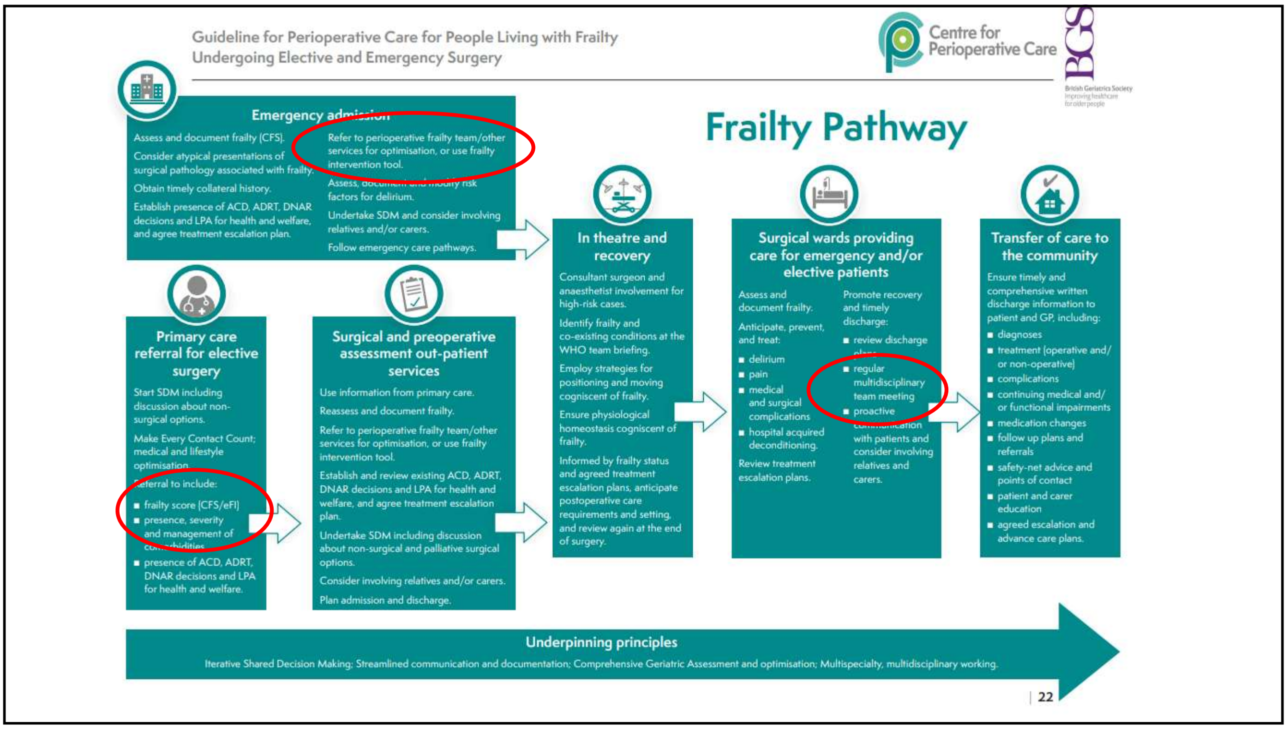

12

Provision of Services for People with Vascular Disease 2021

OFD 1-3 PAD QIF	Safe Effective	**6.5. Medical specialists trained in comprehensive geriatric assessment (CGA) are available to review patients both pre- and post- operatively**	-	-	Self-declaration

Peri-operative care

2.24 Much work has already been done by the Royal College of Anaesthetists and its partners to improve peri-operative medicine. [13]

2.25 VASGBI have produced guidance for both healthcare professionals and for patients on getting the best outcomes from vascular surgery. [14-15]

2.26 People with vascular disease should have access to a comprehensive geriatric assessment (CGA) by a suitably trained specialist to address issues of frailty and multi-morbidity both before and after they have vascular surgery. [16]

6.36 In vascular patients age is not an absolute indicator of need, medical specialists with an interest in the elderly and frail have a pivotal role in using CGA to: [11-13]

- Assess risks of surgery
- Support shared decision making
- Guide pre-operative optimisation
- Advise on post-operative management

6.37 The Centre for Perioperative Care (CPOC) and the British Geriatric Society (BGS) have worked together to develop a whole pathway guide on perioperative care for people living with frailty. [14]

University of Glasgow | College of Medical, Veterinary & Life Sciences
Royal College of Physicians and Surgeons of Glasgow
Vascular Society — The Vascular Society for Great Britain and Ireland
Circulation Foundation — The Vascular Charity

Frailty management

Does your service offer something similar this?

- If not, why?
- If yes, how was it set up?

Do you think there has been a notable improvement in care following implementation?

Is the service used optimally? Can it be improved? Barriers to implementation?

University of Glasgow | College of Medical, Veterinary & Life Sciences
ROYAL COLLEGE OF PHYSICIANS AND SURGEONS OF GLASGOW
VASCULAR SOCIETY
CIRCULATION FOUNDATION The Vascular Charity

Designing a frailty-centric service

If you were to design a frailty-related clinical service models, or adapt established models, for this growing cohort of patients, what would it look like?

University of Glasgow | College of Medical, Veterinary & Life Sciences
ROYAL COLLEGE OF PHYSICIANS AND SURGEONS OF GLASGOW
VASCULAR SOCIETY
CIRCULATION FOUNDATION The Vascular Charity

Round up

Unexplored areas?

Questions?
