# Supplementary figures and images for: Assessing stakeholder’s perception and utilisation of frailty assessment in a vascular surgery setting – a national mixed methods study
Source: BMC Surg. 2026 May 11;26:448. doi: 10.1186/s12893-026-03803-5 (PMC13340002; doi:10.1186/s12893-026-03803-5)

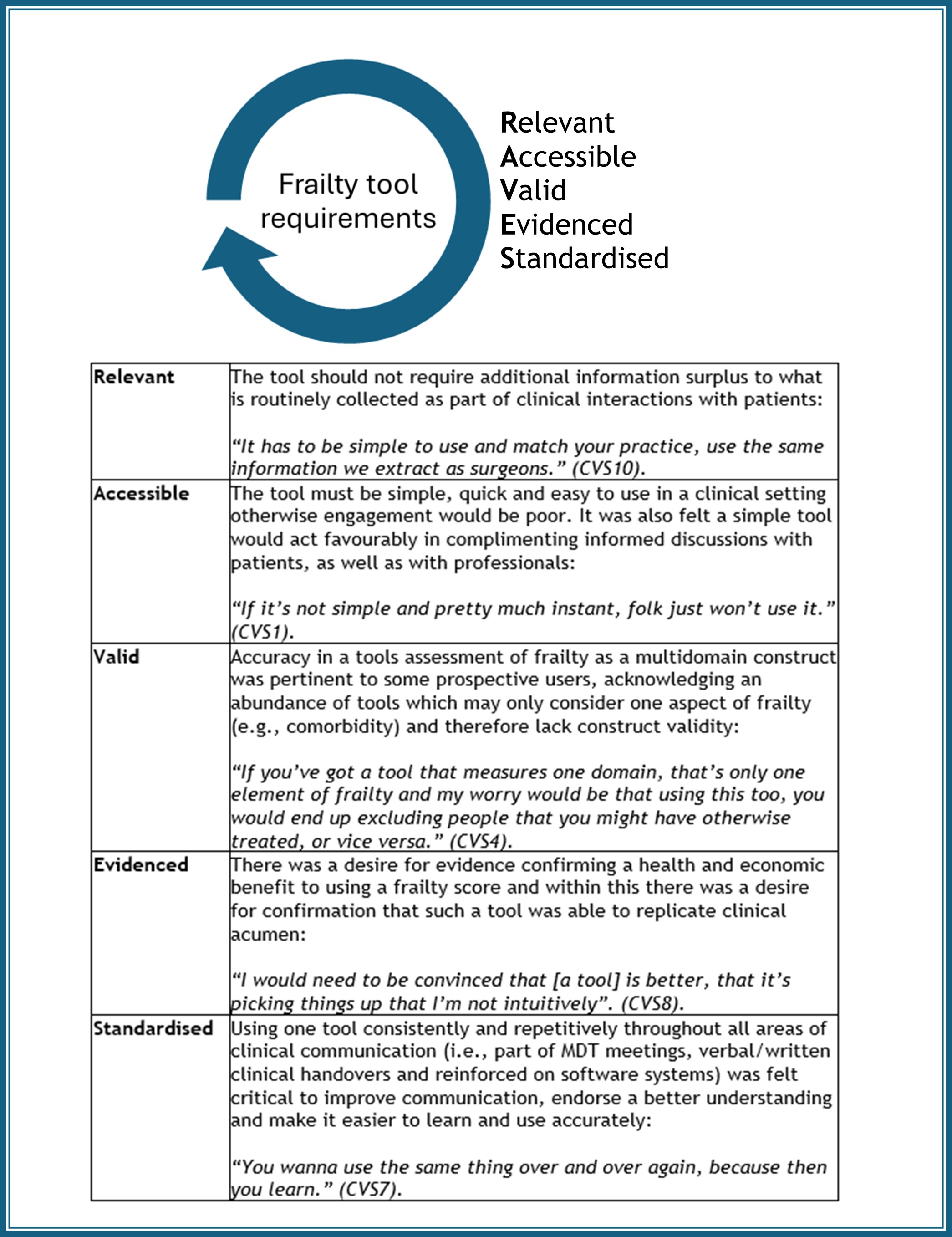

Supplement: Supplementary file 3 — Supplementary Material 3: Supplementary Figure 3 – Desired frailty assessment tool characteristics. [file 12893_2026_3803_MOESM3_ESM.jpg]
