# Supplementary material for: Assessing stakeholder’s perception and utilisation of frailty assessment in a vascular surgery setting – a national mixed methods study
Source: BMC Surg. 2026 May 11;26:448. doi: 10.1186/s12893-026-03803-5 (PMC13340002; doi:10.1186/s12893-026-03803-5)
Supplement: Supplementary file 4 — Supplementary Material 4: Supplementary Table 1 – Interprofessional differences in practice. [file 12893_2026_3803_MOESM4_ESM.docx]

Supplementary Table 1 – Interprofessional differences in practice.

|  | **Surgeons**  **(consultant and trainees)**  ***n*=41 (%)** | **Non-surgeons**  ***n*=19 (%)** | **Consultant vascular surgeons**  ***n*=24 (%)** | **Surgical trainees**  ***n*=17 (%)** |
| --- | --- | --- | --- | --- |
| **Comfortable with the frailty concept (scores 4+/5)** | 40 (98) | 13 (68) | 24 (100) | 16 (94) |
| **Agree added value to assessing frailty over comorbidity (scores 4+/5)** | 32 (78) | 14 (74) | 20 (83) | 12 (71) |
| **Agree added value to assessing frailty over disability (scores 4+/5)** | 34 (83) | 15 (79) | 21 (88) | 13 (77) |
| **Use frailty to guide management plans (scores 4+/5)** | 26 (63) | 4 (21) | 17 (71) | 9 (53) |
| **Use frailty for joint decision making (scores 4+/5)** | 26 (63) | 7 (37) | 18 (75) | 8 (47) |
